# Prospects for the Use of MICP Technology in the Remediation of Saline–Alkaline Soil Heavy Metal Pollution

**DOI:** 10.3390/microorganisms14030681

**Published:** 2026-03-18

**Authors:** Haiyang Guo, Na Wang, Quan Ma, Junshen Wang, Xiaopeng Gao

**Affiliations:** College of Life Sciences, Yan’an University, Yan’an 716000, China; guoocean1999@163.com (H.G.); yxmnnzs@outlook.com (N.W.); m15191134034@163.com (Q.M.); 15891783352@163.com (J.W.)

**Keywords:** MICP, saline–alkaline soil, heavy metal pollution, ureolytic bacteria, bioremediation

## Abstract

Soil salinization and heavy metal pollution represent significant global challenges to farmland sustainability and food security. Globally, over 800 million hectares of land are affected by salinity, with approximately 17% of cultivated land exhibiting concentrations of at least one heavy metal exceeding established agricultural safety thresholds. Microbially Induced Calcium Carbonate Precipitation (MICP) is an innovative biogeochemical process that harnesses microbial metabolic activities to facilitate soil mineralization. The core mechanism involves ureolytic microorganisms hydrolyzing urea to produce carbonate ions (CO_3_^2−^). These ions subsequently react with environmental calcium ions (Ca^2+^) to form insoluble calcium carbonate (CaCO_3_) precipitates. This review synthesizes recent research progress on the application of MICP technology for the remediation of heavy metal pollution. It elucidates the mechanistic pathways by which MICP immobilizes heavy metal ions and critically evaluates its potential application for ameliorating heavy metal contamination specifically within saline–alkaline soils. Key challenges impeding the broader practical deployment of MICP are analyzed, particularly concerning salt-alkali stress tolerance and the management of ammonia emissions during urea hydrolysis. Emerging strategies, such as the synergistic integration of MICP with biochar amendments, offer promising solutions. Biochar can provide a protective microenvironment for microbial consortia and potentially mitigate ammonia volatilization, thereby enhancing the overall efficacy and feasibility of this remediation approach for contaminated saline–alkaline lands.

## 1. Introduction

On 21 October 2021, during the Global Symposium on Salt-affected Soils, the Food and Agriculture Organization of the United Nations (FAO) launched the “Global Map of Salt-affected Soils”. This landmark report identifies factors such as rising sea levels, poor land management practices, inappropriate fertilizer application, deforestation, and seawater intrusion as key drivers of soil salinization [1]. There are over 800 million hectares of salt-affected soils that exist globally [2].

Heavy metals exhibit pronounced toxicity and are prone to bioaccumulation and biomagnification through food chains, posing significant threats to human health [3,4,5]. Their inherent hazardous potential persists at trace concentrations [6]. Chronic cadmium exposure may induce anemia, emphysema, nephrotoxicity, and carcinogenesis [7]. Lead exposure detrimentally impacts neurocognitive development and behavioral patterns in children [8]. Arsenic exposure correlates with elevated risks of malignancies in the lung, skin, bladder, and urinary tract [9,10]. Hexavalent chromium (Cr^6+^) is classified as a respiratory carcinogen, with inhalation exposure augmenting lung cancer incidence [11].

Recent accelerated industrialization has intensified heavy metal contamination in saline–alkaline soils globally. Environmental drivers—including near-surface temperature, precipitation variability, and elevated potential evapotranspiration—contribute to spatially heterogeneous metal accumulation [12]. Excessive application of chemical fertilizers can also lead to the accumulation of heavy metals in saline–alkali soils [13]. Arid and semi-arid regions of Asia, Africa, and the Americas are increasingly subjected to the synergistic pressures of soil salinization and heavy metal contamination. This convergence of abiotic stressors presents cascading threats to agricultural productivity and ecosystem integrity. (Table 1).

MICP harnesses microbial metabolic pathways to drive in situ mineralization in soil matrices. While microorganisms mediate diverse mineral formations—including carbonates, oxides, phosphates, sulfides, and silicates [14]—carbonate precipitation via microbial activity represents the most extensively characterized biomineralization process.

MICP leverages ureolytic microbial metabolism to drive carbonate mineralization. The core biochemical pathway comprises four steps:

(1) Urease catalysis:
  CO(NH_2_)_2_ + H_2_O → 2NH_3_ + CO_2_

(2) Carbonate equilibrium:
  CO_2_ + H_2_O → H_2_CO_3_ → HCO_3_^−^ + H^+^ → CO_3_^2−^ + 2H^+^

(3) Alkalinization:
  NH_3_ + H_2_O → NH_4_^+^ + OH^−^

(4) Precipitation:
  Ca^2+^ + CO_3_^2−^ → CaCO_3_↓

Calcium carbonate crystals can also form a physical barrier around bacteria to immobilize heavy metals, eventually generating stable heavy metal–carbonate complexes [15,16,17]. MICP technology is often applied in engineering fields such as sand fixation, soil stabilization, heavy metal immobilization, crack healing in cement-based materials, CO_2_ sequestration, erosion control, and dust suppression [17,18,19,20]. As an emerging bioremediation method, it has the advantages of being environmentally friendly, low-cost and highly effective in remediation, and is especially suitable for arid regions [21,22].

**Table 1:** Different regions’ soil salinization and the types of hazardous heavy metals.

Area	Soil Property	Types of Heavy Metals	References
Yellow River Delta, China	Saline–Alkaline Soil (pH 7.35–9.17, salt concentration 0.01–2.00%)	As, Cd, Cr, Ni	[23]
Raebareli District, Uttar Pradesh, India	Saline–Alkaline Soil (pH 6.3–9.8, EC 0.24–10.53 μS/cm^3^)	As, Cu, Pb, Zn, Mn	[24]
Murcia, Spain	Saline–Alkaline Soil (pH 6.79–8.47, EC 3.42–17.00 dS m^−1^)	Mn, Zn, Pb, Cd, Cu, As	[25]
Atyrau, Kazakhstan	High-saline soil (salt concentration in dry residue 0.28–0.71%)	Ti, Mn, Mg, Pb, Ni, Cu, Co, Sr, Ag, P, Mo	[26]
Yinda Town, China	Saline–Alkaline Soil	Zn, Pb, Hg	[27]

## 2. Common Heavy Metal Remediation

### 2.1. Physical Remediation and Chemical Restoration

Physical remediation is a process that uses physical means to remove or control pollutants in the soil in order to achieve soil remediation. Common physical remediation methods include soil replacement, soil amendment, physical sieving, and electrokinetic remediation (Table 2). These methods mainly involve replacing heavy metal-contaminated soil with clean soil to effectively reduce the heavy metal content in the soil. Electrokinetic remediation is an advanced technology that utilizes the effect of an electric field to remove heavy metal pollution from soil. Chemical precipitation, leaching, solvent extraction, chemical reduction/oxidation, and other methods are the most common chemical remediation techniques. The current single chemical precipitation technology is difficult to achieve strict environmental standards economically and effectively, and most studies are limited to laboratory scale [28]. Chemical precipitation technology is often used for the removal of heavy metals in industrial wastewater. However, due to the structural characteristics of soil, the chemical precipitation method may not achieve the expected remediation effect in soil.

### 2.2. Bioremediation

#### 2.2.1. Phytoremediation

Phytoremediation involves planting specific heavy metal-tolerant plants, which utilize their roots’ strong absorption and accumulation capabilities to effectively transfer heavy metals from the soil into the plant tissues. Over 500 plant species have been identified that can accumulate heavy metals [50,51]. However, phytoremediation takes a longer time, and plant growth is inhibited in environments with high concentrations of heavy metals, resulting in lower efficiency in removing heavy metals.

#### 2.2.2. Microbial Remediation

Microbial remediation of heavy metal-contaminated soil is a method that uses the biochemical characteristics of microorganisms to reduce the bioavailability and toxicity of heavy metals in the soil. The main pathways for microbial remediation of heavy metal-contaminated soil are as follows: first, through the oxidation-reduction actions of microorganisms, the valence states of heavy metals are changed. Highly toxic valence states of metals are oxidized or reduced into less toxic and more stable valence states [41]. Second, heavy metal forms are transformed by microorganisms [41]. Third, extracellular polymeric substances (EPS) secreted by bacteria remove heavy metal ions from the environment through adsorption, precipitation, or complexation [42]. Shen Guoqiang [43] and others used chitosan microspheres loaded with *Bacillus subtilis* to reduce the solubility of trivalent chromium and decrease the transformation of trivalent chromium to hexavalent chromium, which is beneficial in reducing the toxicity of chromium to plants. Zeng Yong [44] and others confirmed that *Sporosarcina ureilytica* ML-2 played a key role in the transformation of metal forms in cadmium-contaminated sludge, preventing the transfer of ionic Cd from the sludge to the supernatant. The fixation efficiency reached 98.46%, the soluble exchangeable forms of Pb and Cd were reduced by 100% and 48.54%, respectively, and the residual forms of Pb and Cd increased by 22.54% and 81.77%. Priyadarshanee [45] and others studied the adsorption behavior and interaction mechanisms of the EPS of *Pseudomonas aeruginosa* OMCS-1 with chromium (Cr), lead (Pb), and cadmium (Cd), confirming that the interactions between EPS and heavy metals are spontaneous. Microbial remediation is environmentally friendly and sustainable with low cost and low energy consumption. It causes little disturbance to soil structure, fertility and microbial communities, and rarely produces secondary pollution. It is well-suited for in situ remediation of large-scale, low-concentration polluted farmland or sites. The MICP technology induces soil mineralization through the metabolic activities of urease-producing bacteria without the need for additional immobilization. It features high product stability and strong leaching resistance, enabling the long-term safe sequestration of heavy metals. As a mild in situ bioremediation approach, it requires no large quantities of chemical reagents, produces no secondary pollution, and causes minimal disturbance to the soil environment. It can efficiently colonize and function in extreme environments such as saline–alkali soils without drastically altering the soil’s physical and chemical properties, thereby achieving the synergistic goals of heavy metal solidification and saline–alkali soil improvement. Featuring simple processes, low cost, and excellent ecological compatibility, this technology is suitable for the large-scale and sustainable remediation of heavy metal contamination in extensive saline–alkali lands.

### 2.3. Combined Remediation

Compared with single-method remediation, combined remediation can improve remediation efficiency through synergistic effects. It is not limited to a single remediation method and is a flexible remediation approach. It includes a variety of combined approaches, such as phytoremediation–microbe, microbe–organic material, electrokinetic–phytoremediation [47,48,49], and others, and suitable choices can be made according to specific environmental conditions. Liu Yingying [52] and others have used *Hydrocotyle rotundifolia* in combination with *Klebsiella* sp. strains to remediate lead (Pb) pollution in water environments, effectively enhancing the absorption and tolerance of lead by *H. rotundifolia*. Xu Min [49] and others reduced the migration rate of cadmium (Cd) in soil by 23.6% and 45.8% through the construction of a biochar–bacteria partnership, and increased soil fertility, bacterial diversity, and abundance by 11.7–90.2%, 5.4–16.1%, and 6.8–54.7%, respectively. In recent years, combined remediation technology has attracted widespread attention in the field of environmental governance. This technology has significant advantages in removing heavy metal ions, especially in dealing with composite pollution of multiple heavy metal ions, where it demonstrates high remediation efficiency. Among them, the combined remediation technology of plants and microorganisms has shown particularly outstanding performance in the governance of heavy metal pollution, providing new ideas and methods for the ecological remediation of heavy metal-contaminated soils.

## 3. The Basic Principle of Using MICP for the Remediation of Heavy Metals in Saline–Alkali Soils

**Figure 1:**
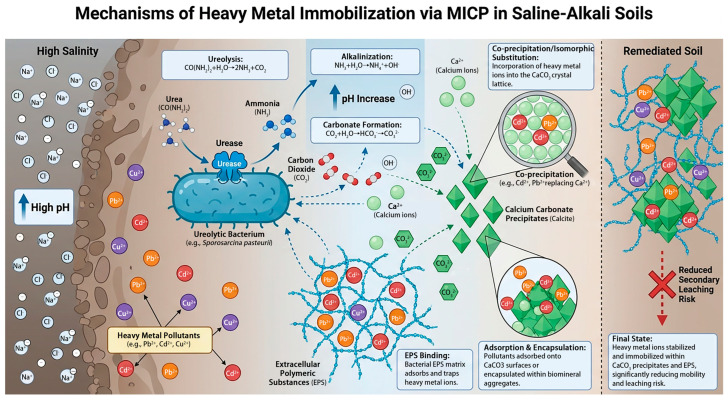
Pathway of heavy metal immobilization by ureolytic bacteria via MICP in high saline–alkaline environment.

As a bioremediation technique, MICP can be used to treat heavy metal pollution in a variety of situations. The MICP process leads to the precipitation of calcium carbonate, which can effectively immobilize toxic metal pollutants. These pollutants can be immobilized through precipitation or co-precipitation without being affected by the metal valence, toxicity, or redox potential [53]. During the MICP process, ureolytic bacteria produce urease, which hydrolyzes urea into ammonium and carbonate, leading to the precipitation of calcium carbonate. Metal ions with ionic radii similar to that of Ca^2+^ may enter the calcium carbonate crystals by replacing Ca^2+^, making the immobilized metal ions difficult to release [54] and slowing down the migration of heavy metal ions into the environment [20,22,55]. The microscopic mechanism of calcium carbonate nucleation during the MICP process is still unclear. The specific hydroxyl density in soil can promote the dispersed deposition of CaCO_3_. A higher concentration of Ca^2+^ can induce rapid and uniform nucleation [56]. The hydroxyl groups in soil provide ion-binding sites, which facilitate rapid nucleation of calcium carbonate. The efficiency of MICP in immobilizing different metal ions varies [57]. This may be related to factors such as the ionic radius and chemical properties of metal ions, the solubility product constants of carbonates formed by different metal ions, and the adsorption capacity of microbial surfaces [58,59]. When Cd^2+^ enters the calcium carbonate lattice, its larger ionic radius allows it to form a more stable structure, making it less susceptible to secondary leaching due to external environmental influences. Ureolytic bacteria mainly immobilize heavy metal ions through the following ways (Figure 1): (1) During the MICP process, CaCO_3_ coatings form on soil particles containing heavy metals. The CaCO_3_ layer protects heavy metal ions from secondary leaching through physical shielding and chemical buffering, significantly enhancing their stability and reducing migration risks in the environment [60]. (2) During the metabolic process of urease-producing bacteria, a large amount of EPS is generated, including various amino acids. Under alkaline conditions, most amino acids carry negative charges due to deprotonation, which allows them to adsorb positively charged metal ions. This promotes the aggregation of heavy metal ions and reduces their mobility in the environment [58]. (3) During the precipitation of calcium carbonate, heavy metal ions can co-precipitate with calcium carbonate or undergo an exchange reaction with the calcium ions in calcium carbonate, entering the crystal lattice structure of calcium carbonate. This transforms heavy metals from a soluble state to an insoluble state, thereby reducing their mobility [61]. In practical field experiments, environmental conditions are complex, and there may be co-contamination by multiple metal ions. In such cases, various immobilization mechanisms will work simultaneously, making it somewhat one-sided to discuss only a single immobilization mechanism.

## 4. The Research Progress of MICP

**Figure 2:**
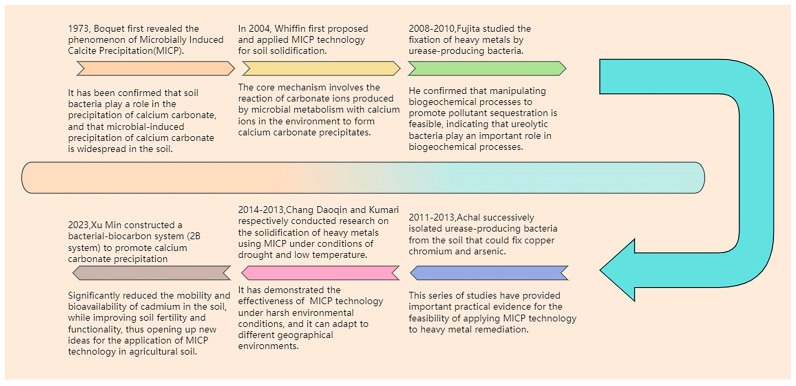
Timeline of MICP technology’s application and development.

After the introduction of MICP technology, researchers began to explore its applications in various fields, such as soil improvement, soil solidification, and heavy metal pollution remediation (Figure 2). In 1973, Boquet [62] and others first revealed the phenomenon of MICP, confirming the role of soil bacteria in calcite precipitation and the widespread occurrence of MICP in soils. In 2004, Whiffin [63] first proposed and applied MICP technology for soil solidification. The core mechanism involves the reaction of carbonate ions produced by microbial metabolism with calcium ions in the environment to form calcium carbonate precipitates.

From 2008 to 2010, Fujita [64,65] conducted research on the immobilization of heavy metal ions using ureolytic bacteria. He performed a preliminary assessment of urea dissolution-driven calcite precipitation and strontium co-precipitation for the remediation of ^90^Sr contamination in the 100-N area of Hanford, Washington. The study confirmed that the site has the biogeochemical characteristics required for the application of ^90^Sr calcite precipitation remediation methods. It also demonstrated the feasibility of manipulating biogeochemical processes to promote the sequestration of pollutants, with ureolytic bacteria playing an important role in this process.

From 2011 to 2013, Achal [66,67,68] used MICP technology to remediate copper, arsenic, and chromium contamination. By employing different urease-producing bacteria in soil amended with 340 mg/kg of copper, the content of exchangeable copper in the soil was significantly reduced from 67 mg/kg to 3.5 mg/kg after 120 h of treatment, achieving a copper removal rate of 98%. The content of exchangeable arsenic in the soil decreased from 25.85 mg/kg to 0.88 mg/kg, while the content of arsenic bound to carbonates increased from 14.7% to 22.3%. In soil amended with 500 mg/kg of Cr^6+^, the content of exchangeable Cr^6+^ decreased from 25.85 mg/kg to 0.88 mg/kg, and the content of Cr^6+^ bound to carbonates increased from 14.7% to 22.3% (Table 3).

In 2014, Kumari [69] and others used *Exiguobacterium undae* to immobilize cadmium in contaminated soil at low temperatures (10 °C). Over 90% of the cadmium in the soil was transformed from the soluble exchangeable fraction to the carbonate-bound fraction. In 2023, Chang Daoqin [70] and others conducted research on the optimal cementation cycles of MICP technology for the remediation of heavy metal-contaminated soils in the northwest region, providing a theoretical basis for the efficient application of MICP technology in the remediation of soil pollution in arid areas. These studies indicate that MICP technology can adapt to different geographical environments and has the potential to be applied in various regions. Omoregie [71] isolated urease-decomposing bacterial colonies from the local landfill site. The removal efficiency of Cd^2+^ was 99.10%, that of Ni^2+^ was 76.33%, that of Cr^3+^ was 26.67%, and that of Cu^2+^ was 17.61%. Comadran-Cas [57] found that after treatment using MICP technology, the removal rate of the exchangeable forms of lead and zinc in the soil exceeded 85%.

Current research on MICP for heavy metal remediation in contaminated soils is constrained by several critical limitations: First, studies often adopt a single-contamination orientation with insufficient strategies for complex pollution. Existing studies predominantly focus on the immobilization of individual heavy metals, exhibiting pronounced metal-specific remediation efficiencies. Targeted regulation and intensification strategies for poorly immobilized metals, particularly in complex multi-heavy-metal co-contamination systems, remain inadequately investigated. Second, current studies demonstrate a weak adaptability to extreme environments with a systematic knowledge gap regarding saline–alkali soils. The applicability of MICP under extreme soil conditions is limited. While current research has extensively addressed low-temperature and arid regions, systematic investigations of MICP remediation in saline–alkali soils characterized by high salinity, elevated pH, and poor structural stability are still absent. The inhibitory mechanisms of salt stress on urease activity, microbial growth kinetics, and CaCO_3_ precipitation dynamics, as well as the long-term stability of heavy metal immobilization under saline–alkali conditions, have yet to be elucidated. Third, studies show insufficient attention to long-term stability and environmental risk assessment. Most studies emphasize short-term immobilization efficiency and heavy metal fraction transformation, whereas research on the long-term stability of carbonate-bound heavy metals under leaching, freeze–thaw cycling, and field environmental fluctuations remains scarce. The potential risks of heavy metal re-release have not been comprehensively evaluated. Fourth, there is a lagging micro-scale mechanistic understanding relative to macroscopic characterization. Current achievements are largely confined to macroscopic chemical fraction transformation (exchangeable fraction → carbonate fraction). Microscopic mechanisms of heavy metal co-precipitation with microbially induced CaCO_3_, including crystal morphology, binding speciation, and sequestration pathways, have received limited attention. Fifth, there is an absence of integrated remediation–amelioration technologies. Present research exclusively emphasizes heavy metal immobilization while neglecting the simultaneous improvement of soil pH, salinity reduction, and fertility enhancement in saline–alkali soils. An integrated technical system combining heavy metal remediation with saline–alkali soil amelioration has not been developed or validated.

## 5. The Combination of MICP and Biochar for the Remediation of Heavy Metal Pollution

**Figure 3:**
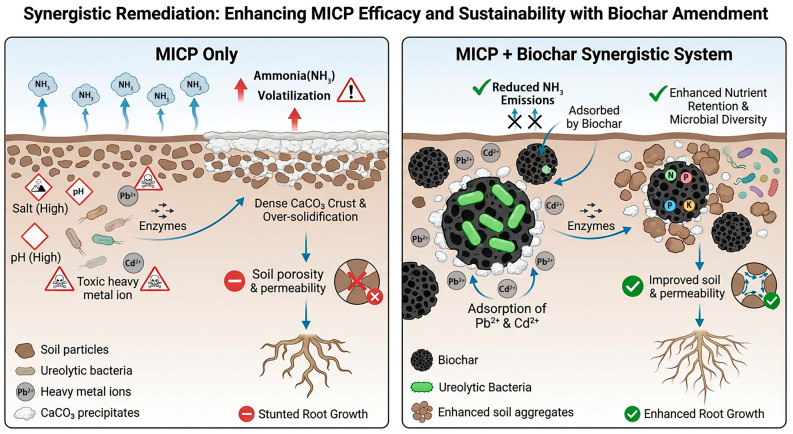
Remediation of heavy metal pollution in highly saline–alkali agricultural land by combining MICP with biochar.

While MICP is highly effective for heavy metal remediation, it frequently triggers concomitant alterations in soil physical properties, such as pronounced aggregation, enhanced mineral crystallization, and increased shear strength, which collectively impede soil porosity and permeability. These alterations may restrict its practical application and large-scale promotion in agricultural soils [19,72,73]. Although the increase in soil strength is advantageous in some engineering applications [57,74], for agricultural soils, however, excessive soil strength may limit the growth and expansion of plant roots, thereby affecting the plants’ absorption of water and nutrients (Figure 3). The effects of MICP on soil structural properties in saline–alkali environments have been demonstrated by Xiong et al. [75], who employed *Sporosarcina pasteurii* ATCC 11859 in low-salinity soils (salt content < 6%). Their findings indicated significant reductions in mean porosity (19–26%) and permeability (62.77%), underscoring MICP’s capacity to modify soil physical architecture under salt-stressed conditions. During the MICP process, the generated calcium carbonate (CaCO_3_) precipitates preferentially deposit at soil particle contact points, pore spaces and micropore regions, which directly fill the primary pores of coarse-grained saline–alkali soils, reduce the total pore volume, and thereby realize an effective reduction in soil porosity. Meanwhile, the CaCO_3_ precipitates, dominated by calcite clusters, form cementation bridges at particle contact points to cement the loose coarse-grained soil particles into an integrated whole, which inhibits the deformation and expansion of pores and further lowers the soil porosity and permeability. Biochar amendments have shown promise in enhancing soil physical and hydraulic properties through their inherent physicochemical characteristics. Moradi-Choghamarani et al. [76] investigated sugarcane bagasse biochar application across three distinct soil orders, revealing divergent structural responses: pore reorganization and altered aeration in loamy Aridisols, and increased inter-aggregate porosity in silty-clay Inceptisols and silty-clay-loam Alfisols. Notably, biochar-treated Inceptisols and Alfisols exhibited a greater volume of drainable pores (VDP), suggesting enhanced water retention and transmission properties. In the study by Xu Min [49] and others, a bacteria–biochar system (2B system) was constructed to promote calcium carbonate precipitation. The biochar provided a protective effect for the bacteria, significantly enhancing soil bacterial diversity and abundance. The 2B system notably reduced the mobility and bioavailability of cadmium in the soil, while also improving soil fertility and functionality. This research has paved the way for the application of MICP technology in agricultural soils. Biochar has an adsorption effect on heavy metals. Xirui Kang and others [77] treated soil with potassium permanganate (KMnO_4_)-hematite-modified biochar (MnFeB), which significantly reduced the DTPA-extractable Zn and Cd contents in the soil by 18.79% and 43.65%, respectively. The porous structure and high specific surface area of biochar provide protection for microorganisms against environmental stress. The organic carbon and mineral nutrients carried on the surface of biochar can serve as electron donors and acceptors for microorganisms, promoting their colonization and proliferation [78]. Although combined remediation shows great promise, existing research is still at the laboratory stage and faces three major bottlenecks: First, there is a lack of specialized strains for saline–alkali–heavy metal co-contamination, and the compatibility between strains and biochar is insufficient. Second, the parameters of combined remediation (such as biochar addition rate and strain inoculation ratio) have not been optimized, resulting in unstable field application effects. Third, the long-term stability of combined remediation needs to be verified. Most studies have only monitored for 3–6 months, and there is a lack of long-term data for over one year. Compared with single microbial remediation, choosing the appropriate combined remediation method may achieve twice the result with half the effort.

## 6. Challenges of Applying MICP Technology to the Remediation of Heavy Metal Pollution in Saline–Alkali Soils

### 6.1. Salt Stress

Heavy metal pollution in saline–alkali soils has become a severe situation. When applying MICP technology to saline–alkaline soil, the first consideration should be whether urease-producing strains can tolerate saline–alkali stress. High salinity has an adverse effect on the richness and biological activity of soil microbial communities [79], and it can inhibit the activity of strains. Screening for strains that are already tolerant to high salinity in saline–alkali soils is very important for the application of MICP technology in the remediation of heavy metals in such soils. Jianmiao Xu [80] and others isolated a strain of high urease activity and boron-tolerant spore-forming bacteria from soil samples. This strain has salt and alkali tolerance. At pH 13, the OD_600_ value can reach 2.0 after 24 h of cultivation; at a salt concentration of 6%, the OD600 value can reach 1.0 after 24 h of cultivation. The most significant effect of high salinity on the MICP process is its impact on the crystal structure of carbonate. Yuze Wang and others found in their study that in distilled water environments, calcium carbonate crystals primarily take on a rhombohedral shape, whereas in high-salinity seawater environments, the crystals form a mushroom-like shape. These mushroom-shaped crystals are less efficient in particle bridging and strength enhancement [81]; Similarly, Jianyu Yang and others observed that in deionized water, the primary crystal shape of calcium carbonate produced by *Sporosarcina pasteurii* is rhombohedral. However, in an environment with a salinity of 3.5%,the predominant shape of calcium carbonate crystals is hemispherical, which affects the final outcome of MICP [82]. This also indicates that in the MICP process in high-salinity environments, unstable aragonite and vaterite are formed. This leads to a reduced rate of heavy metal immobilization and an increased likelihood of secondary leaching. These issues highlight one of the challenges in applying MICP technology to saline–alkali soils.

### 6.2. pH

pH is a crucial factor in the MICP process, as it affects microbial activity, bacterial growth, urease activity, and CaCO_3_ precipitation [83]. An excessively high pH can inhibit the growth of microbial strains. However, urea-decomposing bacteria are capable of adapting to adverse pH conditions [84]. This mechanism inherently leads to an increase in local soil pH during the process [57]. When the initial pH of the culture medium varies between 6 and 10, an alkaline environment leads to more CaCO_3_ precipitation [76]. The study by Jiang Zhaoming [74] and others showed that the immobilization effect of MICP on Cd^2+^ was optimal at a pH of 9, with a removal rate of 97.43%. Gang Zhou et al. [58], in their study using *Bacillus pasteurii* in combination with hydrothermal carbonization to remediate lead ions, found through XRD and XPS analysis of the mineralization products that Pb^2+^ can form not only PbCO_3_ but also Pb(OH)_2_ by combining with OH^−^ in an alkaline environment. Lead hydroxide may form hydrated lead oxide products under alkaline conditions and may undergo hydrolysis in the natural environment, which will also affect the lead immobilization efficiency.

### 6.3. Ammonia Emission

Urea produces ammonia under the action of urease-producing bacteria. The volatilization of ammonia is one of the main pathways for nitrogen loss and has negative impacts on the environment and soil health, including soil acidification, eutrophication of water bodies, and increased greenhouse gas emissions [85]. Urease inhibitors can reduce ammonia volatilization by directly acting on urea, thereby decreasing ammonia loss [86]. However, this contradicts the conditions required for the MICP process. By combining urease-producing bacteria with biochar, the production of ammonia can be appropriately reduced. Khadim Dawar and others [87] found in their study that the combined application of urea and biochar significantly reduced NH_3_ emissions. Compared to the use of urea alone, biochar reduced NH_3_ emissions by 27%. An appropriate concentration of urea and urease activity can control the emission of ammonia. Additionally, the addition of biochar can also help reduce ammonia emissions. In experiments, the optimal urea concentration is explored while ensuring the production of calcium carbonate. By considering the impact of various factors on ammonia emissions, a balance can be achieved between heavy metal pollution remediation and ammonia emissions. Weighing the pros and cons is key to ensuring the sustainable application of MICP in the remediation of heavy metal pollution in saline–alkali soils. Environmental challenges make international cooperation and regulation extremely necessary. By integrating quality assurance, stakeholder participation, and environmental protection through a comprehensive approach [88], effectively combining the upstream policy-making with the downstream scientific research to solve the problem of industrial pollution, this research can progress rapidly.

## Figures and Tables

**Table 2:** Common remediation methods for heavy metal-contaminated soil.

	Physical Remediation	Chemical Restoration	Bioremediation	Combined Remediation
Principle	Physical remediation is a process that uses physical means to remove or control pollutants in the soil in order to achieve soil remediation.	By adding chemical reagents to the soil, the heavy metal ions react with the reagents, causing chemical changes.	Bioremediation is a technology that utilizes the physiological functions and metabolic mechanisms of organisms to achieve the transport, enrichment, and oxidation degradation of heavy metal ions in the soil, thereby effectively reducing their environmental hazards.	Combined remediation is a method for remediating heavy metal pollution by using multiple remediation techniques.
Remediation method	Soil replacement, soil amendment, physical sieving, and electrokinetic remediation.	Chemical precipitation, leaching, solvent extraction, chemical reduction/oxidation.	Phytoremediation, microbial remediation.	Phytoremediation-microbe, microbe-organic material, electrokinetic-phytoremediation and others.
Advantage	Fast treatment speed, intuitive and stable remediation effects, and strong adaptability to high-concentration and heavily polluted sites.	High efficiency and rapid onset, quickly transform heavy metals into stable fractions and reduce their bioavailability effectively.	Environmentally friendly and sustainable with low cost and low energy consumption.	Combined remediation can improve remediation efficiency through synergistic effects; it is not limited to a single remediation method and is a flexible remediation approach.
Disadvantage	Large engineering quantities, high energy consumption and remediation costs, and are difficult to apply to large-scale farmland soil remediation.	Introduce exogenous reagents and cause secondary pollution, strongly affected by soil pH, salinity and other conditions.	Time-consuming with low treatment efficiency, strongly affected by environmental conditions.	--
References	[29,30,31,32,33,34,35]	[28,36,37]	[38,39,40,41,42,43,44,45,46]	[47,48,49]

**Table 3:** The immobilization of heavy metals by various urease-producing bacteria.

Strain	Initial Concentration	The Content of Exchangeable	The Content of Carbonate-Bound State	References
*Sporosarcina ginsengisoli* CR5	500 mg/kg As (III)	25.85 → 0.88 mg/kg	14.7% → 22.3%	[67]
*Bacillus* sp. CS8	500 mg/kg Cr (VI)	124.8 → 2.6 mg/kg	11.2 → 106.4 mg/kg	[68]
*Kocuria flava* CR1	340 mg/kg Cu (II)	67 → 3.5 mg/kg	--	[66]
*Exiguobacterium undae* YR10	100 mg/kg Cd (II)	36.5 → 1.2 mg/kg (10 °C)	21 → 67.8 mg/kg (10 °C)	[69]
*Sporosarcina pasteurii*	2.74 mg/kg Cd	Reduced 23.6%	increase 45.8%	[49]

## Data Availability

No new data were created or analyzed in this study. Data sharing is not applicable to this article.

## Abbreviations

The following abbreviations are used in this manuscript:

2B system	Bacteria-biochar system
DTPA	Diethylenetriaminepentaacetic acid
EPS	Extracellular polymeric substances
FAO	Food and agriculture organization of the united nations
MICP	Microbially induced calcium carbonate precipitation
XPS	X-ray photoelectron spectroscopy
XRD	X-ray Diffraction
